# Surgical treatment of biliary atresia with patent distal extra hepatic bile ducts: Is hepatic portocholecystostomy the right choice?

**DOI:** 10.4103/0971-9261.54817

**Published:** 2009

**Authors:** V. V. S. Chandrasekharam

**Affiliations:** Rainbow Children's Hospital and SVR Hospital, Hyderabad, Andhra Pradesh, India

**Keywords:** Biliary atresia, cholangitis, jaundice, Kasai

## Abstract

**Purpose:**

To report the results of surgical treatment of biliary atresia with patent distal extra hepatic bile ducts (BA with PDEBD) with special reference to hepatic portocholecystostomy (HPC) operation.

**Materials and Methods:**

The study reviews records of children operated for BA with PDEBD. The type of operation, results of surgery, postoperative course and complications during follow-up are noted.

**Results:**

Five children (mean age 83 days) underwent surgery for biliary atresia with patent extra hepatic bile ducts. The diagnosis was confirmed by intraoperative cholangiography in each case. Three children underwent HPC and two had standard hepatic portoenterostomy (HPE) as HPC was not technically feasible. The operation was considered successful in three of five children (60%, two HPC and one HPE), partially successful in one. The mean follow-up was 22 months. None of the children with HPC had cholangitis at follow-up; one child with HPE had recurrent cholangitis.

**Conclusions:**

Biliary atresia (BA) with PDEBD may be a variant with a fair chance for surgical success. When feasible, HPC may be a good treatment option in this group with acceptable results and practically no risk of postoperative cholangitis.

## INTRODUCTION

Hepatic portoenterostomy (HPE) described by Kasai has become the standard operation for noncorrectable biliary atresia (BA).[1] The most common and serious complication of HPE is ascending cholangitis which results in progressive hepatic fibrosis even after the initial success of HPE. Many modifications of Kasai operation have been described with the main aim of minimizing cholangitis, but with limited success.[2] There is a variant of BA with patent distal extra hepatic bile ducts (PDEBD) which accounts for 10-20% of BA cases.[3–5] In this variant, the gallbladder, cystic duct and common bile duct are patent, with atresia of the proximal extra hepatic ducts. Hepatic portocholecystostomy (HPC) has been recommended as an alternative procedure in this variant of BA.[3,4,6–8] HPC is the modification of Kasai operation with the least risk of cholangitis. There are not many reports on the results of operative treatment of PDEBD variant of BA. Although available literature suggests that HPC is a good option in this subgroup of patients,[4,6,7] a recent report indicates that there may be problems specific to the HPC in the long-term.[5] In this paper, we review the results of surgery in five cases of BA with PDEBD, including HPC in three of the five cases. To our knowledge, this is the first Indian report on this technique.

## MATERIALS AND METHODS

Five cases of BA with PDEBD were managed (group A) from February 2002 to September 2007. During this period, the author managed a total of 34 cases of BA, 29 being the garden variety (type 3) of BA (group B). Thus, the PDEBD variant accounted for about 15% of BA cases (five of 34) managed during that period by the author. The patency of distal ducts in all the five cases was confirmed by intraoperative cholangiography. Three of these five patients underwent HPC; the gallbladder was very small with precarious vascularity in the other two children. Hence, it was decided to abandon HPC and do a standard HPE operation. Clearance of jaundice, cholangitis and other problems were recorded on follow-up. Postoperatively, the achievement of jaundice-free status (serum bilirubin level less than two mg %) was considered to indicate a successful operation. Postoperative pigmented stool with reduction in bilirubin levels (but still more than two mg %) was considered a partial success. No change in stool color was taken to indicate a failed operation. All children received standard postoperative medication which included phenobarbitone, ursodeoxycholic acid, prophylactic antibiotics and steroids for three months.

## RESULTS

The comparison between the two groups is given in Table 1. The initial bile drainage was similar in both groups while success (serum bilirubin less than two mg/dl) was achieved in 60 and 44% in groups A and B respectively. These differences between the groups did not reach statistical significance due to small number of patients in group A. The mean follow-up in group A is 22 months (range six-50). Of the three children who underwent HPC operation, two (66%) became jaundice-free; the third child who underwent HPC saw partial success with periods of pigmented and nonpigmented stool and a bilirubin value that remained greater than two mg%. At the most recent follow-up of 24, 12 and 50 months respectively, one child remains jaundice-free while the other two are asymptomatic with a serum bilirubin of 3.0mg% (direct bilirubin 0.4, indirect 2.6 indicating good bile drainage but with liver damage) and 2.4 mg% (direct 1.9, indirect 0.5). None of the three children who underwent HPC had postoperative cholangitis while 31% (seven of 22) of children who drained bile after HPE had at least one episode of severe cholangitis requiring hospitalization. Of the two children with PDEBD who underwent standard HPE operation, one (50%) became jaundice-free within one month postoperatively. However, he had repeated episodes of cholangitis and now at 18 months follow-up has serum bilirubin 2.3 mg% with deranged liver function tests. The other child with PDEBD who underwent HPE had a failed operation and is being considered for a liver transplant.

**Table 1:** Comparison of the two groups

Groups	Total no. of cases	Initial bile drainage post op (%)	Success (serum Bil <2 mg%)	Cholangitis
Group A (mean age 83 days)	5 (3 HPC, 2 HPE)	4 (80)	3(60)	0 of 3 HPC
Group B (mean age 79 days)	29 (HPE)	21 (74)	13 (44)	7 of 22 HPE (31%)

## DISCUSSION

To our knowledge, there are no previous reports from our country on the use of HPC operation in BA with PDEBD. Considering the advanced patient age at surgery in this series, the results of surgical treatment of BA with PDEBD are encouraging. It is known that the chances of a successful Kasai operation reduce significantly after 60 days of age, so that in children older than 60 days, only limited success may be expected.[9] However, in a country like India, where liver transplantation is not yet well established, a successful Kasai operation is the only way to prolong survival in children with BA. Even in countries with a successful liver transplant program, Kasai operation is still considered the first choice treatment for BA.[10] In our series of the five cases of BA with PDEBD, Kasai operation was successful in three children (60%; two HPC, one HPE) with partial success in one child. This may indicate that PDEBD variant of BA generally has a reasonably good chance of surgical success. In the present series, HPC operation had acceptable results without significant postoperative problems over a mean follow-up period of about two years. There was no episode of cholangitis in the HPC group. In contrast, in the HPE group, 31% children had cholangitis.

The most significant complication after a successful Kasai operation is cholangitis.[9,11] Cholangitis not only adds to the morbidity but also results in progressive liver damage. Many modifications of Kasai operation have been described to prevent cholangitis, but with limited success.[2] Some of them actually added significantly to the morbidity and are rarely used now. HPC was described by Kasai in 1974 as an excellent operative method to prevent postoperative cholangitis in children with BA with PDEBD.[3] Lilly later reported on four cases of HPC in BA with PDEBD. Two of the cases showed excellent results, while the other two had to be converted to bilioenteric anastomosis due to obstruction of the distal ductal system.[4] He recommended that HPC was a feasible surgical option in BA with PDEBD and an effective operation to prevent cholangitis. Other authors also supported the role of HPC in preventing cholangitis in BA with PDEBD.[12]

The major criticism against HPC is that the patent distal ducts in PDEBD are narrow and may gradually get blocked with time which may require revision to intestinal conduit.[5] Matsuo *et al*. observed that the patent distal duct in BA with PDEBD had a significantly smaller diameter than a normal duct.[5] These authors postulated that due to the small diameter these ducts were prone to blockage and other problems in the long-run. However, other available reports on the long-term results of HPC do not seem to support this. It was observed by Lilly that there was a gradual increase in diameter of the distal bile ducts after HPC.[8] It is likely that the unused ducts were small initially but increased in size after the operation once bile flow was established through them. This might explain why all cases of HPC did not have problems with distal duct obstruction. Valayer reported excellent results in 15 children with HPC who survived for over 10 years after the surgery, without liver transplantation.[6] Karrer *et al*. also reported on six long-term survivors after HPC[7] and Nio *et al*. reported that two children survived over 20 years after HPC.[13] In our series, we have not had procedure-related problems in any of the three children who underwent HPC.

It is noteworthy that in two of the five children in our series, HPC was attempted but had to be abandoned in favor of HPE. This was because the gallbladder and distal bile duct could not be dissected and mobilized with satisfactory preservation of their vascularity. It is likely that persistence with HPC in these two children may have resulted in failure of HPC and/or subsequent complications. There were no specific features on the cholangiography to suggest that these two patients would be unsuitable for HPC. Hence, we would venture an HPC in all such cases and abandon it in favor of HPE only based on intraoperative observations of the gallbladder vascularity and viability after mobilization.

In conclusion, BA with PDEBD may be a variant with a fair chance of surgical success. When feasible, HPC may a good treatment option in this group, with acceptable results and practically no risk of postoperative cholangitis.
